# Identifying the risks associated with tissue expander infections among patients undergoing mastectomy following clinic-based expansion procedures

**DOI:** 10.1017/ash.2026.10325

**Published:** 2026-03-27

**Authors:** Madeline Ngo, Courtney Koplyay, Andrew Y. Zhang, Tessy Davidson, Roman Jandarov, Jeffrey Konnert, Madhuri M. Sopirala

**Affiliations:** 1 UT Southwestern Medical School: The University of Texas Southwestern Medical Center, USA; 2 Department of Plastic Surgery, University of Texas Southwestern Medical Center, USA; 3 Parkland Hospital: Parkland Health, USA; 4 University of Cincinnati College of Medicine, USA; 5 Infectious Diseases/Internal Medicine, UT Southwestern: The University of Texas Southwestern Medical Center, USA

## Abstract

Case control analysis of breast tissue expander (TE) infections after clinic-based expansion procedures from 2019 to 2022 in a large county hospital found no significant modifiable risk factors, including implant type. Suboptimal sterile access may be an independent contributor to TE infections following clinic procedures. Ongoing protocol adherence and monitoring are needed.

## Introduction

Implant-based breast reconstruction accounts for approximately 80% of breast reconstructions in the United States.^1,2^ Two-stage reconstruction involves placement of a tissue expander (TE) mastectomy, followed by serial clinic-based expansions with permanent implant placement or autologous reconstruction at a later date. Tissue expansion requires repeated needle access of the TE and is associated with increased risk of infection.^3^ TE infections remain the leading cause of readmission and expander loss after immediate implant-based reconstruction, delay second-stage reconstruction, imposing a great socioeconomic burden on the patient.^4–7^

In response to staff reports of increased postTE expansion infections after adoption of a new implant type in October 2021, we conducted a retrospective surveillance analysis of patients undergoing clinic-based expansion after mastectomy. In 2020, the postTE expansion infection rate at our institution was 5.3%. The objectives of this study were to identify risk factors associated with TE infections following TE manipulation, and to assess infection risk associated with two commonly used FDA-approved TE implants.^8,9^ Implant Type 1 uses a single access port, whereas implant type 2 has a dual-port system for expansion and periprosthetic fluid aspiration. Existing literature does not demonstrate a difference in infection rates between implant types. However, infection risk following clinic-based expansion procedures has not been previously studied.

## Methods

We conducted a retrospective review of all adult female patients who underwent mastectomy with immediate TE placement from 2019–2022 at a large, safety net institution (n = 292). Variables studied are listed in Table 1. Postoperative antibiotic prophylaxis was prescribed at the discretion of the surgical team. Peri-operative prophylaxis was standardized per institutional protocol and was not evaluated as a variable in this analysis. Cases and controls were identified through retrospective review of electronic medical records. Cases were defined as patients who underwent mastectomy with immediate TE placement, had at least one clinic-based expansion procedure, and developed an expansion-related TE infection within a 90-day surveillance period after the first expansion.

**Table 1.** Univariable analysis of potential risk factors for tissue expander infection after clinic-based expansion, 2019–2022

Risk factor	TE infection (n = 17) n (%)	No TE infection (n = 212) n (%)	*P*-value
Race			
White	14 (82.4%)	157 (74.4%)	0.7201
Black	3 (17.6%)	51 (24.1%)	
Asian	0 (0%)	3 (1.4%)	
Ethnicity			
Non-Hispanic	4 (23.58%)	64 (30.2%)	0.7624
Hispanic	13 (76.5%)	148 (69.8%)	
Spanish speaking			
No	7 (41.2%)	86 (40.6%)	1
Yes	10 (58.8%)	126 (59.4%)	
Private insurance			
No	13 (76.5%)	201 (94.8%)	0.015
Yes	4 (23.5%)	11 (5.2%)	
Medicaid			
No	11 (64.7%)	165 (77.8%)	0.3494
Yes	6 (35.3%)	47 (22.2%)	
Medicare			
No	16 (94.1%)	200 (94.3%)	1
Yes	1 (5.9%)	12 (5.7%)	
Hospital financial assistance			
No	10 (58.8%)	136 (64.2%)	0.8591
Yes	7 (41.2%)	76 (35.8%)	
Uninsured			
No	16 (94.1%)	196 (92.5%)	1
Yes	1 (5.9%)	16 (7.5%)	
Surgical wound class			
Clean	14 (82.4%)	166 (78.3%)	0.9931
Clean-Contaminated	3 (17.6%)	24 (11.3%)	
Missing	0 (0%)	22 (10.4%)	
American society of anesthesiologists (ASA) rating			
1	0 (0%)	9 (4.2%)	0.5727
2	9 (52.9%)	121 (57.1%)	
3	8 (47.1%)	80 (37.7%)	
Diabetes			
No	14 (73.7%)	193 (86.5%)	0.3048
Yes	3 (15.8%)	19 (8.5%)	
Implant type			
Implant 1	9 (52.9%)	173 (82.0%)	0.0106
Implant 2	8 (47.1%)	38 (18.0%)	
Doxycycline prophylaxis			
No	9 (52.9%)	131 (61.8%)	0.6442
Yes	8 (47.1%)	81 (38.2%)	
Cephalexin prophylaxis			
No	7 (41.2%)	121 (57.1%)	0.3094
Yes	10 (58.8%)	91 (42.9%)	
Clindamycin prophylaxis			
No	16 (94.1%)	211 (99.5%)	0.3409
Yes	1 (5.9%)	1 (0.5%)	
Trimethoprim-sulfamethoxazole Prophylaxis			
No	17 (100.0%)	176 (83.0%)	0.1325
Yes	0 (0%)	36 (17.0%)	
Ciprofloxacin prophylaxis			
No	16 (94.1%)	208 (98.1%)	0.8242
Yes	1 (5.9%)	4 (1.9%)	
Immunocompromised			
No	11 (64.7%)	130 (61.3%)	0.9865
Yes	6 (35.3%)	82 (38.7%)	
Human immunocompromised virus (HIV)			
No	17 (100.0%)	209 (98.6%)	1
Yes	0 (0%)	3 (1.4%)	
Chemotherapy			
No	10 (58.8%)	134 (63.2%)	0.921
Yes	7 (41.2%)	78 (36.8%)	
Dialysis			
No	17 (100.0%)	211 (99.5%)	1
Yes	0 (0%)	1 (0.5%)	
Renal disease			
No	17 (100.0%)	208 (98.1%)	1
Yes	0 (0%)	4 (1.9%)	
Hypertension			
No	9 (52.9%)	161 (75.9%)	0.0721
Yes	8 (47.1%)	51 (24.1%)	
Cardiovascular disease			
No	16 (94.1%)	195 (92.0%)	1
Yes	1 (5.9%)	17 (8.0%)	
Obesity			
No	11 (64.7%)	120 (56.6%)	0.6929
Yes	6 (35.3%)	92 (43.4%)	
Tobacco use disorder			
No	13 (76.5%)	181 (85.4%)	0.5276
Yes	4 (23.5%)	31 (14.6%)	
BMI mean (SD)	29.65 (5.11)	29.44 (4.88)	0.8724
Procedure duration (hours) mean (SD)	3.76 (1.44)	3.72 (1.34)	0.8933

BMI, Body mass index; SD, Standard deviation.

Percentages are column percentages (within TE infection and no TE infection groups).

Denominators may vary by variable due to missing data.

Table includes patients undergoing clinic-based expansion between 2019–2022.

Immunocompromised: receipt of chemotherapy, chronic immunosuppressive therapy, or HIV positive.

Obesity: BMI ≥ 30 kg/m^2^.

Renal disease: chronic kidney disease of any stage.

Controls were defined as patients who underwent mastectomy with immediate TE placement, at least one clinic-based expansion procedure, and reconstruction but did *not* develop a TE infection. The unit of analysis was the patient rather than the individual breast or TE. Postoperative infections were identified based on imaging findings, culture results, and supporting symptoms (fever, erythema, tachycardia, leukocytosis, purulent drainage, and presence of seroma). Retrospective surveillance started in December 2021. Expansion-related TE infections were classified using Centers for Disease Control and Prevention/National Health Safety Network (CDC/NHSN) criteria^10^ and included deep incisional and organ/space infections temporally associated with clinic-based expansion procedures. Superficial incisional infections and surgical-site infections occurring prior to the initiation of expansion were excluded. Data were recorded utilizing Research Electronic Data Capture (REDCap). Due to the small number of infection events, analyses of temporal trends were descriptive and not intended as formal time-series inference.

In response to surveillance findings and staff reports of increased infections following clinic-based expansion, direct observations of TE access procedures were conducted in the plastic surgery clinic beginning in February 2022. Observed opportunities for improvement included variability in sterile field setup and skin preparation, inconsistent disinfection of port-site magnet locators, and reuse of procedure trays between patients. These observations informed development of a standardized sterile access protocol, which included alcohol-based chlorhexidine gluconate skin preparation with increased surface area of skin prepped, use of sterile drape, and expanded sterile field, disinfection of magnet locators between patients, and use of a 3-way stopcock for saline installation.

Categorical variables were compared using χ^2^ or Fisher’s exact tests where appropriate. Continuous variables were compared using Student’s *t*-test. Stepwise logistic regression analysis (entry criterion 0.05) was used to identify factors associated with TE infections. For multivariable logistic regression, cases were analyzed with a randomly selected 1:3 ratio of controls. No matching was performed. Data analysis was conducted using SAS software version 9.4.

The project was deemed as quality improvement by an institutional review process and the need for research approval was waived.

## Results

Of 292 patients included between 2019–2022, sixteen (5.5%) developed a TE infection after clinic-based expansion. The majority of patients were Spanish speaking (56.2%). Only 7.7% of patients had private insurance, while the remaining had Medicaid (23.3%), Medicare (7%), county funded financial assistance (34.5%), other (33.1%), or were uninsured (7.3%).

**Figure 1.**
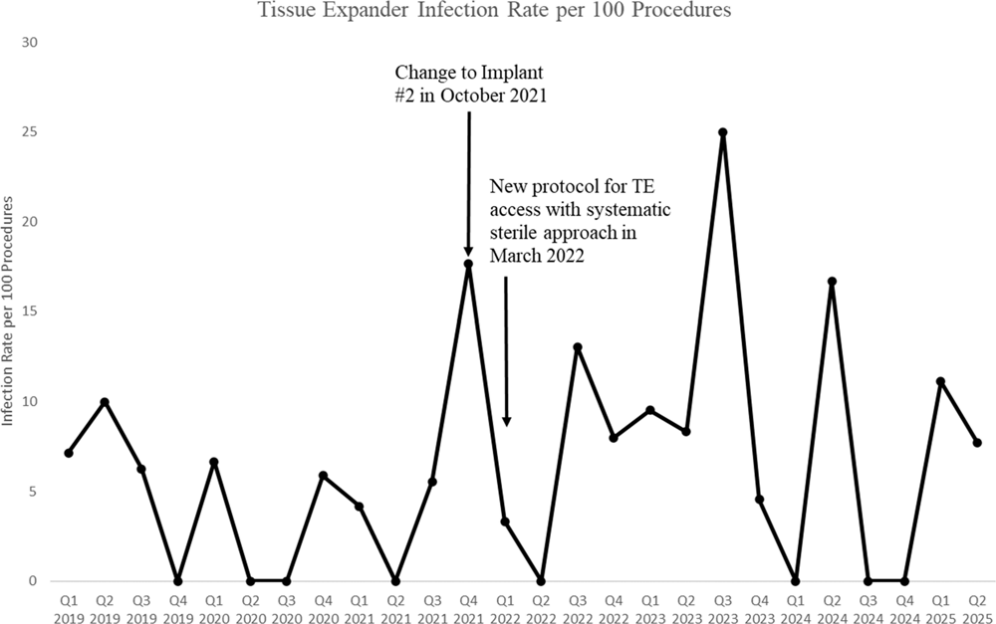
Temporal trends in tissue expander infection rates following implant change and sterile access protocol implementation.

Visual trend analysis demonstrated a spike in TE infections following the introduction of Implant #2 in October 2021. Infection rates subsequently declined after implementation of a new sterile access protocol in March 2022 (Figure 1). As shown in Table 1, patients receiving Implant 2 had a higher infection rate than those receiving Implant 1 (17.4% vs 5.0%, *P* = .0106). There were no other modifiable risk factors associated with increased rates of TE infections following TE manipulation. On multivariate analysis, the use of Implant Type 2 (vs Implant Type 1) was associated with increased odds of TE infections (OR 2.14, 95% CI 0.62–7.41), though this association did not reach statistical significance (*P* = .232) (Table 2). Having private insurance was significantly associated with an increased risk of TE infection. Patients with private insurance had over six times the odds of developing an infection compared to those without private insurance (OR 6.12, 95% CI 1.55–24.13; *P* = .01). Infection rates declined following protocol implementation but demonstrated variability over subsequent quarters.

**Table 2.** Multivariate analysis of modifiable risk factors

Risk factor	Odds ratio	95% Confidence interval	*P* value
Hypertension	2.52	[0.83, 7.66]	.103
Private insurance	6.12	[1.55, 24.13]	.010
Implant 2	2.14	[0.62, 7.41]	.232

## Discussion

To our knowledge, this is the first study to report infection rates and risk factors associated with clinic-based TE expansion procedures. Adoption of Implant Type 2 occurred beginning in October 2021 and did not represent an immediate or complete replacement of Implant Type 1. Visual trend analysis suggests that infections increased following the introduction of Implant Type 2, and declined after standardizing sterile technique. However, subsequent spikes observed in Q3 2023 and Q2 2024 suggest that this reduction was not uniformly sustained and may reflect variability in protocol adherence, unmeasured factors, or random fluctuation due to small sample size. Formal auditing of long-term protocol adherence was not performed, highlighting the need for sustained quality oversight. Prior studies evaluating TE infections have primarily focused on operative and patient-level risk factors, including obesity, diabetes, smoking, radiation therapy, and peri-operative antibiotic practices, with limited attention to clinic-based expansion procedures. Our approach aligns with prior SSI and TE infection studies while focusing our analysis on clinic-based expansion procedures. While not statistically significant in multivariate analysis, Implant Type 2 was associated with increased odds of infection compared with Implant Type 1. This may warrant further investigation in a larger cohort. Private insurance was independently associated with higher infection risk. This counterintuitive finding may reflect unmeasured confounding or case selection bias.

This study has several limitations. As a retrospective, single-center analysis, infections may have been missed among patients lost to follow-up or treated elsewhere. Age was not analyzed due to the narrow age range of the cohort (30–65 years) and the number of expansion procedures was unavailable. Generalizability may be limited given institutional variability in protocols. The small number of infection events limited statistical power to detect significant differences between implant types. Multicenter studies or registry-based analyses are needed to better characterize infection risk related to TE design and access techniques.

## References

[1] Broyles JM , Balk EM , Adam GP et al. Implant-based versus autologous reconstruction after mastectomy for breast cancer: a systematic review and meta-analysis. Plast Reconstr Surg Glob Open 2022;10:e4180. 35291333 10.1097/GOX.0000000000004180 PMC8916208

[2] American Society of Plastic Surgeons. 2018 plastic surgery statistics report. 2018. https://www.plasticsurgery.org/documents/News/Statistics/2018/plastic-surgery-statistics-full-report-2018.pdf. Accessed March 10, 2026.

[3] Bertozzi N , Pesce M , Santi P , Raposio E. Tissue expansion for breast reconstruction: methods and techniques. Ann Med Surg (Lond) 2017;21:34–44. 28765784 10.1016/j.amsu.2017.07.048 PMC5526469

[4] Urquia LN , Henderson SP , Farewell JT et al. Tissue expander-based breast reconstruction at a major safety-net hospital: managing the outsized risk of infection. Aesthet Surg J Open Forum 2022;4:ojac036. 35673613 10.1093/asjof/ojac036 PMC9167491

[5] Cohen JB , Carroll C , Tenenbaum MM , Myckatyn TM. Breast implant-associated infections: the role of the national surgical quality improvement program and the local microbiome. Plast Reconstr Surg 2015;136:921–929. 26505698 10.1097/PRS.0000000000001682

[6] Ozturk CN , Ozturk C , Soucise A et al. Expander/Implant removal after breast reconstruction: analysis of risk factors and timeline. Aesthetic Plast Surg 2018;42:64–72. 29270693 10.1007/s00266-017-1031-8

[7] Sue GR , Sun BJ , Lee GK. Complications after two-stage expander implant breast reconstruction requiring reoperation: a critical analysis of outcomes. Ann Plast Surg 2018;80:S292–S294. 29489547 10.1097/SAP.0000000000001382

[8] Crook J , Farewell J , Zhang A , Kislevitz M. Short term complication profiles of Mentor Artoura vs Sientra AlloX2 tissue expanders for immediate breast reconstruction. Plastic surgery the meeting. 2023. Austin, TX. Abstract #38993.

[9] Parmeshwar N , Piper M , Viner J , Foster R , Kim EA. Evaluation of dual-port versus single-port tissue expanders in postmastectomy breast reconstruction. Plast Reconstr Surg Glob Open 2021;9:e3703. 34367849 10.1097/GOX.0000000000003703 PMC8341374

[10] Centers for Disease Control and Prevention. Surgical site infection (SSI) event. 2024. 11–15. https://www.cdc.gov/nhsn/pdfs/pscmanual/9pscssicurrent.pdf. Accessed March 10, 2026.

